# Successful Vaginal Delivery during Acute Small Bowel Obstruction: A Case Report and Review of the Literature

**DOI:** 10.1155/2021/6632495

**Published:** 2021-03-04

**Authors:** Angela J. Stephens, Stephen M. Wagner, Beth L. Pineles, Eleazar E. Soto

**Affiliations:** Department of Obstetrics, Gynecology and Reproductive Sciences, McGovern Medical School at The University of Texas Health Science Center at Houston (UTHealth), Houston, TX, USA

## Abstract

Small bowel obstruction during pregnancy is rare and can be detrimental to both mother and fetus. In most cases, management eventually involves surgical intervention. Little is known regarding optimal mode of delivery in those with bowel obstruction during pregnancy. We present a case of vaginal delivery during acute small bowel obstruction as well as a review of recent literature regarding mode of delivery in the setting of bowel obstruction. Our case and literature review demonstrates that in pregnancies complicated by small bowel obstruction, successful vaginal delivery can be achieved in those with stable maternal-fetal status.

## 1. Introduction

Small bowel obstruction during pregnancy is rare [1–6]. It is typically due to adhesive disease from previous surgeries [6–11]. Recognition can be difficult as it may mimic symptoms common in normal pregnancy [3, 6, 9, 12–39]. However, recognition is important as bowel obstruction can be detrimental to both the mother and fetus [5, 13, 40]. In most cases, management involves surgical intervention [6]. Little is known regarding optimal mode of delivery in those with bowel obstruction during pregnancy. Here, we present a case of successful vaginal delivery during acute small bowel obstruction as well as a review of recent literature regarding mode of delivery in the setting of bowel obstruction. Written informed consent was obtained from the patient for publication of this case report and accompanying images.

## 2. Case Presentation

**Figure 1:**
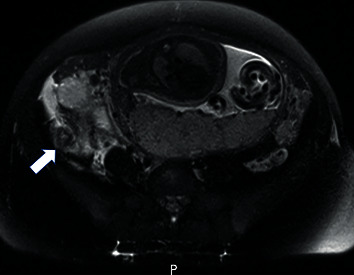
Acute appendicitis detected on T2-weighted MRI image. Dilated appendix (arrow) in the right lower quadrant with surrounding inflammatory changes.

A 20-year-old G1P0 at 29 5/7 weeks presented with abdominal pain, lower back pain, dysuria, and persistent emesis. The patient had been seen two days prior and diagnosed with gastroenteritis. On exam, the patient was noted to be afebrile, tachycardic to 120 beats/minute, and had bilateral costovertebral angle tenderness. Laboratory findings were notable for a leukocytosis of 21 (K/CMM). The patient was diagnosed with pyelonephritis and admitted for intravenous antibiotic administration (ceftriaxone). On hospital day 2, the patient's tachycardia persisted and her pain migrated to the right lower quadrant. A renal ultrasound was negative, and a pelvic magnetic resonance imaging (MRI) demonstrated acute appendicitis (Figure 1). General surgery performed a laparoscopic appendectomy with intraoperative findings notable for a perforated appendix, confirmed by pathology.

Postoperatively the patient received a course of piperacillin-tazobactam and metronidazole. The patient also experienced delayed return of bowel function and persistent nausea and emesis. Computed tomography (CT) obtained for evaluation on postoperative day (POD) 6 confirmed a high-grade bowel obstruction. She was initially managed conservatively with nothing by mouth (NPO), aggressive intravenous hydration, and electrolyte repletion. However, on POD 7, the patient reported right upper quadrant (RUQ) pain and was noted to have a transaminitis with an ALT 609 (U/L) and an AST of 436 (U/L). A RUQ ultrasound noted cholelithiasis and biliary sludge. On hospital day 9 (POD 8), the patient had worsening transaminitis and was scheduled for a laparoscopic cholecystectomy. Laparoscopic findings were notable for adhesions and dilated, friable loops of bowel consistent with large bowel obstruction. Therefore, the case was converted to a laparotomy through a supraumbilical midline vertical incision. The bowel was decompressed via tube decompression, and adhesions were lysed. Pathology confirmed acute and chronic cholecystitis. Following her second surgery, the patient had a delayed return of bowel function but was subsequently discharged home on hospital day 18 (POD 16 and 7).

**Figure 2:**
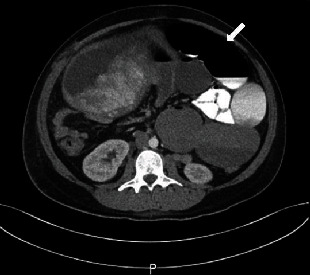
Small bowel obstruction demonstrated on CT. Severely dilated proximal small bowel (arrow) measuring up to 6 cm in diameter with abrupt tapering of the small bowel in the mid abdomen posterior to the dome of the uterus.

She represented to the hospital at 35 4/7 weeks with diffuse abdominal pain and was found to have a partial small bowel obstruction. After consultation with general surgery, the patient was managed conservatively with NPO status, intravenous hydration, and electrolyte repletion. She had a return of bowel function and was discharged home on hospital day 2. One week later, at 36 4/7 weeks, the patient represented with abdominal pain, nausea and emesis, and lack of a bowel movement for several days. She was again diagnosed with a small bowel obstruction confirmed on CT (Figure 2). Given that her vital signs were stable, the decision was made to proceed with induction of labor after consultation with maternal-fetal medicine and general surgery. She had an uncomplicated vaginal delivery of a 2920 gram male infant with Apgar scores of 9 and 9. She remained on bowel rest following delivery, and her diet was slowly advanced. On postpartum day 2, she had a bowel movement and was discharged home on postpartum day 3.

## 3. Materials and Methods

PubMed, Google Scholar, Medline Plus, and Cochrane Review were searched for the timeframe of January 2010 to July 2020. One reviewer screened and extracted data from eligible publications. The keywords and phrases “bowel obstruction in pregnancy” or “bowel obstruction during pregnancy” or “bowel obstruction” or “bowel obstruction” or “pregnancy” were utilized. Included publications were case reports, case series, and reviews with a case presentation component. The exclusion criteria were as follows: non-English language, articles pertaining to nonpregnant patients, or publications related to pregnant patients without bowel obstruction. Data extracted included the following: year of publication, characterization of bowel obstruction at delivery (acute versus resolved), etiology of bowel obstruction, method of bowel obstruction management, gestational age at time of delivery, indication for delivery, and mode of delivery.

## 4. Results

In our review, there was a total of 54 cases of small bowel obstruction during pregnancy from a total of 32 sources during the included timeframe (Tables 1 and 2). Eight of these sources, corresponding to 28 cases, did not discuss the mode of delivery [2, 11, 17, 21, 25, 30, 32, 41]. Of the total 26 cases of small bowel obstruction from 24 sources during pregnancy with a specified mode of delivery, 65.4% (17/26) were ultimately delivered via cesarean delivery. Of the total reviewed cases of small bowel obstruction during pregnancy, 13 were delivered with an active bowel obstruction, and 84.6% (11/13) of these were delivered via cesarean delivery. Nine of thirteen of these patients (9/13 or 69.2%) were delivered concomitant with surgery for the management of bowel obstruction. The majority of cesarean deliveries during acute obstructions were due to fetal or maternal compromise (69.2% or 9/13). Vaginal delivery has also been reported in cases of acute bowel obstruction; 15.4% (2/13) of those with active bowel obstruction were delivered via vaginal delivery. Of the 13 cases of remote bowel obstruction in concomitant pregnancy, 46.2% (6/13) were delivered via cesarean delivery and 53.8% (7/13) were delivered vaginally.

In reviewed cases of bowel obstruction during pregnancy with specified gestational age at delivery, the majority were delivered in the third trimester (92.0% or 23/25). Only 8.0% (2/25) were delivered in the second trimester. None had delivery in the first trimester. Those delivered during an acute bowel obstruction were more likely to be delivered premature. 91.7% (11/12) of those with specified gestational age at delivery were delivered premature as compared to those delivered following resolution of bowel obstruction (7.7% or 1/13). The primary etiologies of bowel obstruction were surgical adhesions (38.4% or 10/26) or volvulus (23.0% or 6/26). Forty percent (4/10) of those with surgical adhesions had a vaginal delivery as compared to 33.3% (2/6) of those with volvulus.

## 5. Discussion

Small bowel obstruction in pregnancy is rare [1–6]. It occurs in roughly 1 in 17,000 deliveries [7]. Although typically caused by adhesions from previous surgeries, other etiologies include hernias, volvulus, malignancy, and appendicitis [6–11]. Recognition of small bowel obstruction in pregnancy can be difficult as symptoms (abdominal pain, nausea, and constipation) are common in normal pregnancies [5, 8, 12, 13, 40, 42]. Delayed diagnosis can result in bowel strangulation and can be detrimental to both the mother and fetus [5, 13, 40]. Studies suggest that maternal mortality and fetal loss in cases of bowel obstruction during pregnancy range from 2-4% to 13-17%, respectively [1, 41, 43, 44].

The management of bowel obstruction during pregnancy is dependent upon several factors including etiology, clinical presentation, maternal-fetal status, and gestational age. Conservative therapy comprising bowel rest, fluid resuscitation, and electrolyte repletion is often the preferred initial approach to care [3, 6, 9, 14]. However, a review of literature demonstrates that surgery is eventually necessitated in the majority of small bowel obstructions during pregnancy [6]. As such, the current literature offers guidance in general approach to bowel obstruction in pregnancy. In contrast, there is limited information regarding obstetric management in pregnancies complicated by bowel obstruction, particularly as it pertains to delivery mode.

A review of literature from 2010 to 2020 (Tables 1 and 2) demonstrates that the majority of women with bowel obstruction during pregnancy are ultimately delivered via cesarean delivery. Of the 26 cases of small bowel obstruction with specified mode of delivery, 65.4% (17/26) were delivered via cesarean delivery. This is particularly true when women are delivered at the time of acute bowel obstruction [6, 7, 9, 12–39]. Thirteen of reviewed cases were delivered with an active bowel obstruction, and 84.6% (11/13) of these were delivered via cesarean delivery, typically due to fetal compromise at the time of concomitant surgery. However, as in our case, this review demonstrates that vaginal delivery has been reported in cases of acute bowel obstruction with 15.4% (2/13) of those with active bowel obstruction being delivered vaginally. However, both of these cases were precipitous deliveries. The likelihood of cesarean delivery in gravidas with bowel obstruction in concomitant pregnancy was decreased if delivered remote from surgery, as in our case [3, 6, 9, 12–39]. Of the 13 cases of remote bowel obstruction in concomitant pregnancy, 46.2% (6/13) were delivered via cesarean delivery and 53.8% (7/13) were delivered vaginally.

Importantly, there appeared to be no difference in delivery mode dependent upon etiology of bowel obstruction. In our reviewed cases, the primary etiologies of bowel obstruction were surgical adhesions and volvulus. Forty percent (4/10) of those with surgical adhesions had a vaginal delivery as compared to 33.3% (2/6) of those with volvulus. Likewise, as with our patient, those delivered during acute bowel obstruction were more likely to be delivered premature. Of those with specified gestational age at delivery, 91.7% (11/12) of those with acute bowel obstruction were delivered premature as compared to 7.7% (1/13) following resolution of bowel obstruction. Given that the majority of those delivered remote from obstruction were term (92.3% or 12/13), there is insufficient data to determine if gestational age alone impacted mode of delivery in those with acute versus resolved bowel obstruction. Similarly, given that the majority of patients required surgical management (88.5% or 23/26), it was difficult to determine if management type impacted the mode of delivery.

As in women with uncomplicated pregnancies, women with bowel obstruction could benefit from vaginal delivery as compared to cesarean delivery. The maternal benefits of vaginal delivery include avoidance of abdominal surgery, decreased risks of infection, hemorrhage, thromboembolism, and shortened recovery period [45].Avoidance of major surgery and a prolonged recovery could be particularly significant in this population as their pregnancy has already been burdened by the physical and psychosocial stressors associated with surgery and the recovery process [46]. Cesarean delivery following bowel obstruction is also likely to be riskier due to adhesions related to previous surgeries, bleeding, infection, or inflammation [47–50]. Additionally, there is often a concern pertaining to the risk of incisional herniation following abdominal surgery. One recent study suggests that cesarean delivery may be associated with increased rates of hernia as compared to vaginal delivery [51]. Finally, there are also neonatal benefits to vaginal delivery including decreased neonatal intensive care unit admissions, need for oxygen resuscitation, and jaundice [47]. In the acute setting, fetal hypoxia associated with bowel ischemia may warrant urgent cesarean delivery. However, outside of this setting, we contend that vaginal delivery should be favored over cesarean delivery for the above reasons.

## 6. Conclusion

Small bowel obstruction in pregnancy is uncommon. There is little guidance regarding the mode of delivery in pregnancies complicated by bowel obstruction. We propose that in clinically stable patients without signs of fetal compromise and who are currently candidates for medical management with nothing by mouth (NPO), aggressive intravenous hydration, and electrolyte repletion, vaginal delivery should be the preferred delivery mode as it optimizes both maternal and fetal outcomes.

## Figures and Tables

**Table 1:** Summary of cases with delivery during acute small bowel obstruction from 2010 to 2020.

	Year of publication	Gestational age at SBO diagnosis (in weeks)	Etiology of SBO	Surgical management?	Gestational age at delivery (in weeks)	Indication for delivery
*Active SBO* *SVD*						
Sherer et al. (case 1)	2016	27	Surgical adhesions	Yes	27	Precipitous delivery
Pereira et al.	2010	33.6	Surgical adhesions	Yes	33.6	Precipitous delivery
*Cesarean delivery*						
Abebe et al.^∗^	2019	32	Appendicitis	Yes	32	Preeclampsia
Metcalfe et al.^∗^	2017	36	Compression from gravid uterus	Yes	36	Compression from gravid uterus in multifetal gestation
Ossendorp et al.^∗^	2016	33	Malignancy	Yes	33	Surgical optimization
Mortelmans et al.	2016	33	Surgical adhesions	Yes	33	Nonreassuring fetal status
Sherer et al. (case 2)^∗^	2016	34	Surgical adhesions	Yes	34	Preeclampsia
Ahmed et al.^∗^	2015	26.5	Sigmoid volvulus	Yes	26.5	Nonreassuring fetal status
Webster et al. (case 1)	2015	39	Surgical adhesions	Yes	39	Nonreassuring fetal status
Wong et al.^∗^	2014	35	Congenital falciform ligament defect with compression from gravid uterus	Yes	35	Nonreassuring fetal status
Zachariah et al.^∗^	2014	29	Surgical adhesions	Yes	29	Maternal compromise
Dracini et al.^∗^	2012	Unspecified	Cecal volvulus	Yes	Unspecified	Fetal demise
Nascimento et al.^∗^	2012	33	Sigmoid volvulus	Yes	33	Fetal demise

^∗^Delivery concomitant with surgical management of bowel obstruction.

**Table 2:** Summary of cases with delivery following resolution of small bowel obstruction from 2010 to 2020.

	Year of publication	Gestational age at SBO diagnosis (in weeks)	Etiology of SBO	Surgical management?	Gestational age at delivery (in weeks)	Indication for delivery
*Resolved SBO* *SVD*						
Nagata et al.	2019	15	Meckel's diverticulum	Yes	40	Term
Constanthin et al.	2017	28	Volvulus of small intestine and surgical adhesions	Yes	40	Term
Kosai et al.	2015	29	Meckel's diverticulum and jejunal volvulus	Yes	30	Premature labor
Bourdin et al.	2014	9	Phytobezoar	Yes	Term	Term
Porter et al.	2014	29	Surgical adhesions	Yes	37	Term
Ekanem et al.	2011	18	Teratoma	Yes	Term	Term
Kang et al.	2011	30	Intussusception	No	37	Term
*Cesarean delivery*						
Kannan et al.	2018	21	Hernia	Yes	40	Term
Daimon et al.	2016	25	Surgical adhesions	No	37	Prior myomectomy
Webster et al. (case 2)	2015	27	Surgical adhesions	Yes	30	Preeclampsia
Serra et al.	2014	28	Hernia	No	Term	Term
Vassiliou et al.	2012	21	Volvulus	Yes	39	Elective
Katawala et al.	2011	20	Hernia	Yes	Term	Nonreassuring fetal status

## Data Availability

Our conclusion is supported by our presenting case as well as the references as listed in the attached tables. All references are readily accessible online.
